# Melatonin protects blood-brain barrier integrity and permeability by inhibiting matrix metalloproteinase-9 via the NOTCH3/NF-κB pathway

**DOI:** 10.18632/aging.102537

**Published:** 2019-12-07

**Authors:** Weiwei Qin, Jing Li, Rongjia Zhu, Suhua Gao, Junfen Fan, Mingrong Xia, Robert Chunhua Zhao, Jiewen Zhang

**Affiliations:** 1 Department of Neurology, State Key Clinical Specialty of the Ministry of Health for Neurology, Henan Provincial People’s Hospital, School of Clinical Medicine, Henan University, Zhengzhou, Henan, China; 2 Institute of Basic Medical Sciences Chinese Academy of Medical Sciences, School of Basic Medicine Peking Union Medical College, Center of Excellence in Tissue Engineering Chinese Academy of Medical Sciences, Beijing Key Laboratory (No. BZO381), Beijing 100005, China; 3 Department of Scientific Research and Discipline Construction, Henan Provincial People’s Hospital, Zhengzhou, Henan, China

**Keywords:** blood-brain barrier, pericytes, matrix metalloproteinase-9, nuclear factor κB, NOTCH3

## Abstract

The pathophysiological mechanism of white matter hyperintensities of cerebral small vessel disease (CSVD) includes an impaired blood-brain barrier (BBB) with increased permeability. Neuroinflammation likely contributes to the disruption of the BBB in CSVD. Therefore, understanding the molecular mechanism of how neuroinflammation causes BBB damage is essential to preventing BBB disruption in CSVD. Matrix metalloproteinase 9 (MMP-9) contributes to BBB damage in neuroinflammatory diseases. In this study, we observed that interleukin-1β (IL-1β)-induced MMP-9 secretion in pericytes increased BBB permeability to sodium fluorescein (Na-F) by damaging the disruption of VE-cadherin, occludin, claudin-5, and zonula occludin-1 (ZO-1). Melatonin reduced BBB permeability to Na-F and inhibited the disruption of the adherens and tight junction proteins. Melatonin also downregulated *MMP-9* and upregulated tissue inhibitor of metalloproteinases 1 (*TIMP-1*) gene expression, which decreased the MMP-9/TIMP-1 ratio. In addition, nuclear translocation of NF-κB/p65 induced by IL-1β in pericytes upregulated MMP-9 expression, which was inhibited by the NF-κB inhibitor PDTC. However, the NOTCH3 inhibitor DAPT significantly inhibited NF-κB/p65 translocation to the nucleus, while melatonin in combination with DAPT significantly prevented NF-κB/p65 translocation than DAPT alone. Our results suggest that melatonin reduced MMP-9-induced permeability of the BBB. Melatonin reduced MMP-9 expression and activity, which was induced by IL-1β through the regulation of the NOTCH3/NF-κB signaling pathway in pericytes, suggesting that pericytes regulate BBB integrity and function.

## INTRODUCTION

Cerebral small vessel disease (CSVD) refers to a syndrome caused by pathophysiologic changes of cerebral microcirculation, including cerebral small arteries, arterioles, venules, and capillaries. CSVD is the main cause of clinical stroke and the leading cause of both silent stroke and cognitive impairment [1, 2]. White matter hyperintensities (WMH) are common magnetic resonance imaging (MRI) feature of CSVD [3]. The pathophysiological mechanism of WMH is the breakdown and increased permeability of the blood-brain barrier (BBB) [4, 5].

The BBB is composed of endothelial cells, astrocytes, and pericytes in the central nervous system (CNS) [2]. Pericytes are chiefly involved in the regulation of blood flow, maintenance of BBB functional integrity, and stabilization of vessel architecture [6–11]. Disruption of the BBB is a key feature associated with neuroinflammatory conditions in CSVD [1]. Matrix metalloproteinases (MMPs), which regulate the structure and function of extracellular matrix molecules under both normal and pathophysiological conditions, are an important mediator of BBB damage [12].

MMPs are a family of zinc-dependent endopeptidases that degrade the basement membranes and extracellular matrix of surrounding cells [13–15]. In brain tissues, MMP-2 and MMP-9 are major contributors to BBB damage. More MMP-9 is secreted from brain pericytes than from the BBB endothelial cells and astrocytes, suggesting that pericytes are the major source of MMP-9 in the CNS [12, 13, 16].

Pericytes are an important cell type in the pathophysiological processes of BBB disruption, including neuronal degenerative changes during age-dependent cognitive impairment disorders [6], such as Alzheimer’s disease (AD) [17, 18], Parkinson’s disease (PD) [19, 20], multiple sclerosis (MS) [21], and cerebral autosomal-dominant arteriopathy with subcortical infarcts and leukoencephalopathy (CADASIL) [22–24]. Pericytes function similarly in all of these disorders, which include BBB dysfunction, and may be affected by the presence of neuroinflammatory molecules [25]. In addition, brain microvascular pericytes exhibit multipotent stem cell characteristics and can differentiate into astrocytes, neurons, and oligodendrocytes in the presence of bFGF [26, 27]. In the neuroinflammatory response that occurs in many neurological diseases, MMP-9 degrades components of the basal lamina, leading to the disruption of the BBB [12]. Pericytes have also attracted attention as neuroinflammatory mediators in the brain [28, 29]. Moreover, IL-1β has been shown to play a vital role in the process of neuroinflammation in the neurological pathologies [30]. IL-1β may be a critical mediator of MMP-9 activity and subsequent disruption of the tight junction in intracerebral hemorrhagic models [31]. Many studies have indicated that IL-1β-induced MMP-9 is secreted from pericytes via the NF-κB signaling pathway [28, 32–34]. Moreover, IL-1β is involved in tongue squamous cell carcinoma and human dystrophic myogenesis via NOTCH signaling pathways [35, 36]. Therefore, we hypothesized that BBB permeability was disrupted by IL-1β inducing MMP-9 secretion from pericytes.

The NOTCH pathway is an evolutionarily conserved signaling system involved in cellular physiology processes of developing and differentiated tissues [37, 38]. There are currently four known NOTCH receptors (NOTCH 1-4) in the vertebrates [39]. Each NOTCH receptor plays a different role in physiological processes; Relevant to CSVD, *NOTCH3* mutations are the critical causative gene defect in CADASIL, which is an autosomal dominant stroke disorder that results in vascular dementia [40] and is the most frequently inherited type of CSVD [41]. CADASIL presents with small arterial vascular smooth muscle cells and brain capillary pericytes [42, 43] that are enriched with NOTCH3 receptors compared with endothelial cells and astrocytes [44]. However, the pathophysiological role of NOTCH3 in brain pericytes remains largely unknown. NOTCH3 regulates multiple NF-κB activation pathways and enhances the release of NF-κB transcription factors from inhibitory components as well as the transacting activity upon downstream promoters [45, 46]. NF-κB is a family of transcription factors that includes p50, p52, p65, RelB, and c-Rel [47]. A heterodimer of p65 and p50 subunits was the first NF-κB molecule described and is inhibited by the IκBα protein in unstimulated cells [48]. NF-κB is activated by TNF-α in human dermal fibroblasts, which in turn can activate pro-MMP-2 [49].

Melatonin is a hormone secreted from the pineal gland and released into the cerebrospinal fluid and circulation. In the brain, melatonin binds to melatonin receptors where it exerts various important biological effects [50–52]. Melatonin demonstrates neuroprotective effects in models of cerebrovascular diseases through either antioxidant function, anti-inflammatory effects, or inhibition of MMPs. Melatonin plays a pivotal role in oxidative and apoptotic signaling, mitochondrial homeostasis, and calcium excitotoxicity in neurodegeneration. Melatonin may affect the NOTCH signaling pathway through multiple targets involved in the pathogenesis of familial and idiopathic PD and regulate leucine-rich repeat kinase 2 (LRRK2), which is a key molecule involved in PD [53]. The melatonin signaling pathways that protect the integrity of the BBB against MMP-9 disruption are currently unknown. Here, we hypothesize that melatonin reduces the expression and activity of MMP-9 secretion in pericytes by regulating the NOTCH3/NF-κB signaling pathway in the BBB. Our hypothesis suggests that pericytes are involved in the regulation of BBB integrity and function and that melatonin is important for BBB protection.

## RESULTS

### Characterization of cells in the triple co-culture BBB model by immunofluorescence

**Figure 1:**
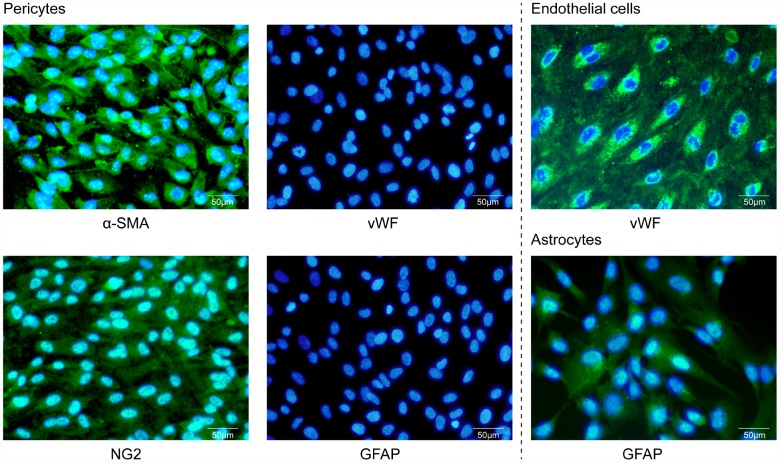
**Characterization of primary cultures by immunofluorescence microscopy.** Human brain microvascular pericytes showed positive immunostaining for α-SMA and NG2 but were negative for endothelial or astrocytic markers. Endothelial cells expressed the von Willebrand factor (vWF), while astrocytes were positive for GFAP. Scale bar = 50 μm.

Prior to the triple co-culture BBB model experiments, the human brain microvascular pericytes, endothelial cells, and astrocytes were characterized. The shape and size of human brain microvascular pericytes were different from the other two cell types of the BBB. Pericytes in culture spread large with irregular projections and were positive for pericyte-markers α-smooth muscle actin (α-SMA) and NG2 chondroitin sulfate proteoglycan and negative for von Willebrand factor (vWF) and glial fibrillary acidic protein (GFAP) staining (Figure 1). Human brain microvascular endothelial cells grew in non-overlapping, continuous monolayers and were large, homogenous, closely opposed and polygonal cells with an oval, centrally located nucleus and indistinct cell borders, and were positive immunostaining for an endothelium marker von Willebrand factor (vWF) (Figure 1). Astrocytes were polygonal with long cell processes and characterized by GFAP immunostaining (Figure 1). These results indicated that the in vitro BBB model is composed of cells that can be differentiated by cell-type-specific morphology and protein expression.

### Melatonin inhibited the expression levels of MMP-9 in pericytes

**Figure 2:**
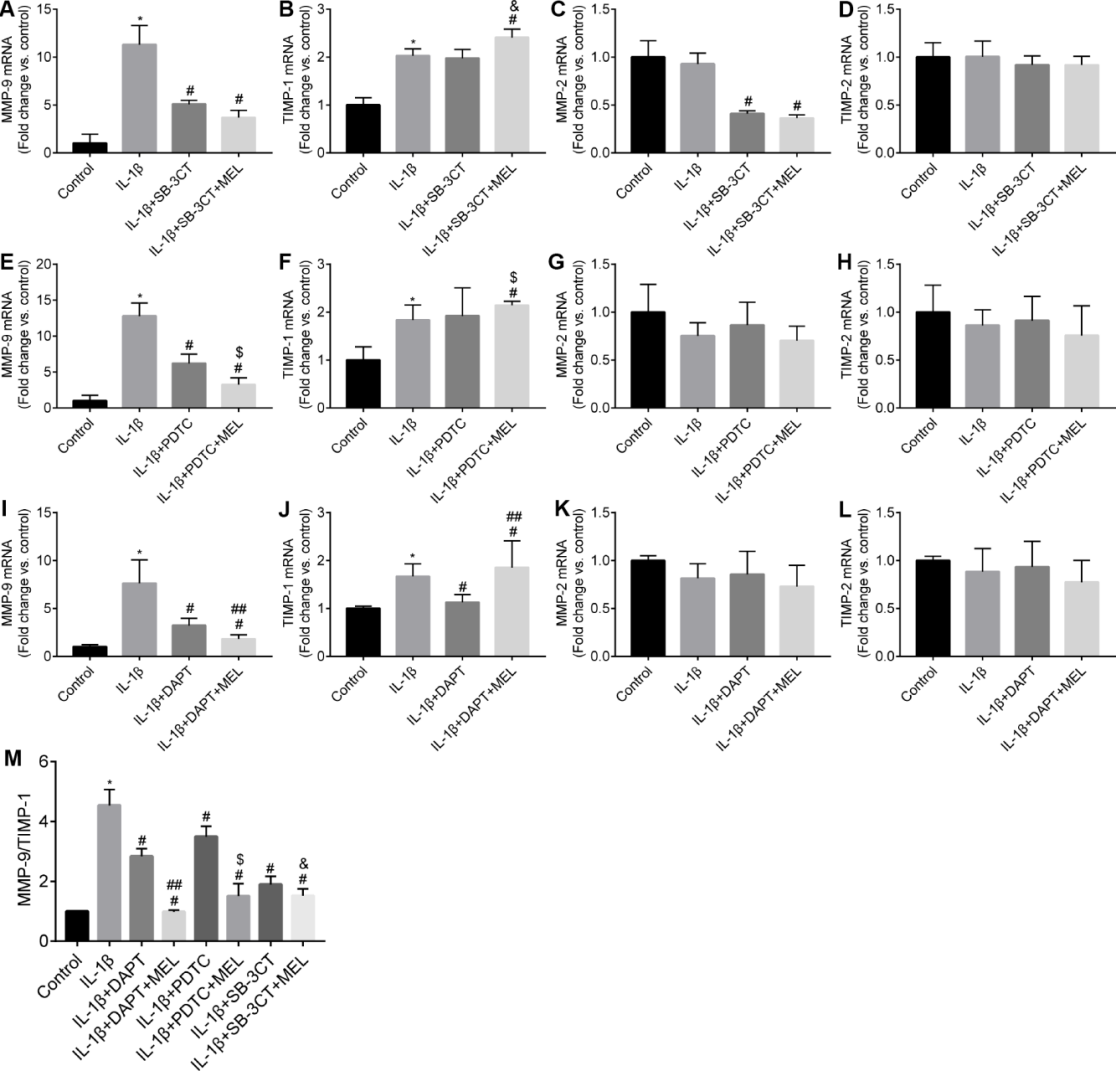
**Melatonin inhibited *NOTCH3* and *MMP-9* but not *MMP-2* gene expression, and increased *TIMP-1* but not *TIMP-2* gene expression in different culture conditions.** Pericytes cultured in the Transwells were pretreated with IL-1β (10 ng/mL) for 24 h in serum-free medium with or without additional pretreatment with DAPT (10 μmol/L), PDTC (25 μmol/L), or WB-3CT (20 μmol/L), and in combination with melatonin (MEL, 10 μmol/L) for 30 min. (**A**–**E**) *NOTCH3*, *MMP-2*, *MMP-9*, *TIMP-1*, *TIMP-2* gene expression levels were quantified in IL-1β-treated, IL-1β + DAPT, and IL-1β + DAPT + MEL groups compared with the control group. qRT-PCR data were normalized to *GAPDH* expression. (**F**–**J**) *NOTCH3*, *MMP-2*, *MMP-9*, *TIMP-1*, *TIMP-2* gene expression levels were quantified in IL-1β-treated, IL-1β + PDTC and IL-1β + PDTC + MEL groups compared with the control group. (**K**–**O**) *NOTCH3*, *MMP-2*, *MMP-9*, *TIMP-1*, *TIMP-2* gene expression levels were quantified in IL-1β-treated, IL-1β + SB-3CT and IL-1β + SB-3CT + MEL groups compared with the control group. (**P**) The ratio of *MMP9/TIMP1* relative transcript levels. Results are presented as the mean ± SD, n = 3. ^*^p < 0.01 compared with the control; ^#^*p* < 0.01 compared with the IL-1β-treated group; ^##^*p* < 0.01 compared with the IL-1β + DAPT group; ^$^*p* < 0.01 compared with the IL-1β + PDTC group; ^&^*p* < 0.01 compared with the IL-1β + SB-3CT group.

We investigated whether pericytes, versus astrocytes and endothelial cells, were the primary producer of MMP-9, (Supplementary Figure 1). The effect of melatonin on the expression of *MMP-9, MMP-2, TIMP-1 and TIMP-2* in pericytes was examined by qRT-PCR. The results showed that *MMP-9* mRNA was higher in the IL-1β-treated group relative to the control group (Figure 2A, 2E, 2I). Upon pretreatment with the MMP-9/-2 inhibitor SB-3CT, the enhanced *MMP-9* expression induced by IL-1β treated was significantly reduced in the IL-1β + SB-3CT group compared with the IL-1β-treated group (Figure 2A). Upon pretreatment with melatonin, the enhanced *MMP-9* expression in the IL-1β-treated group was significantly reduced compared with that in the IL-1β + SB-3CT and IL-1β-treated groups, respectively (Figure 2A). These results suggest that melatonin decreases the IL-1β-induced expression of *MMP-9* in pericytes.

The expression of *MMPs* could be regulated by NF-κB. When the NF-κB inhibitor PDTC was added, the IL-1β-enhanced expression of *MMP-9* in the IL-1β + PDTC group was significantly reduced relative to that in the IL-1β-treated group (Figure 2E). Upon pretreatment with melatonin, *MMP-9* expression was more significantly reduced in the IL-1β + PDTC + MEL group compared with the IL-1β + PDTC and IL-1β-treated groups, respectively (Figure 2E). In addition, NOTCH3 signaling has been predicted to modulate *NF-κB* expression. When the NOTCH3 inhibitor DAPT was added, the IL-1β-induced expression of *MMP-9* was significantly reduced relative to that in the IL-1β-treated group (Figure 2I). Upon pretreatment with melatonin, the enhanced expression of MMP-9 was significantly more reduced in the IL-1β + DAPT + MEL group compared with that in the IL-1β + DAPT and IL-1β-treated groups, respectively (Figure 2I).

We observed no difference in *MMP-2* mRNA level in IL-1β-treated group relative to the control group (Figure 2C, 2G, 2K), which suggested that the *MMP-2* expression was unaffected by IL-1β. When the MMP-9/-2 inhibitor SB-3CT was added to the cell cultures, the expression of *MMP-2* was significantly reduced in the IL-1β + SB-3CT group compared with the IL-1β-treated group (Figure 2C). Upon pretreatment with melatonin, the expression of *MMP-2* was not affected in the IL-1β + SB-3CT + MEL group compared with the IL-1β + SB-3CT group; however, its expression significantly decreased compared with that in the IL-1β-treated group (Figure 2C), suggesting that melatonin did not affect the expression of *MMP-2* in the pericytes.

When the NF-κB inhibitor PDTC was added to cell cultures, there was no difference in the expression of *MMP-2* between the IL-1β + PDTC group and the IL-1β-treated group (Figure 2G). Upon pretreatment with melatonin, the expression of MMP-2 was also not different among the IL-1β + PDTC + MEL, IL-1β + PDTC, and IL-1β-treated groups (Figure 2G). When the NOTCH3 inhibitor DAPT was added, *MMP-2* expression was not different between the IL-1β + DAPT and IL-1β-treated groups (Figure 2K); pretreatment with melatonin also had no effect on *MMP-2* expression among the IL-1β + DAPT + MEL, IL-1β + DAPT, and IL-1β-treated groups (Figure 2K).

TIMP-1 is the endogenous inhibitor of MMP-9; *TIMP-1* mRNA was slightly upregulated in the IL-1β-treated group compared with the control group (Figure 2B, 2F, 2J), but there was no difference between *TIMP-1* expression in the IL-1β + SB-3CT and IL-1β-treated groups (Figure 2B). *TIMP-1* mRNA was significantly upregulated in the IL-1β + SB-3CT + MEL group compared with the IL-1β + SB-3CT and IL-1β-treated groups, respectively (Figure 2B). We observed significant upregulation of *TIMP-1* mRNA in the IL-1β + PDTC + MEL group compared with the IL-1β + PDTC and IL-1β-treated groups, respectively (Figure 2F). However, *TIMP-1* expression was significantly downregulated in the IL-1β + DAPT group compared with the IL-1β-treated group (Figure 2J), while it was significantly more upregulated in the IL-1β + DAPT + MEL group compared with the IL-1β + DAPT and IL-1β-treated groups, respectively (Figure 2J). No difference was observed in *TIMP-2* mRNA levels among the control, IL-1β-treated, IL-1β + DAPT, IL-1β + DAPT + Mel, IL-1β + PDTC, IL-1β + PDTC + Mel, IL-1β + SB-3CT, and IL-1β + SB-3CT + Mel groups (Figure 2D, 2H, 2L).

Finally, we observed that DAPT decreased the effect of IL-1β on the *MMP-9/TIMP-1* mRNA ratio, and melatonin significantly decreased the effect of IL-1β by in the IL-1β + DAPT + MEL group compared with that in the IL-1β + DAPT group (Figure 2M). Melatonin alone or in combination with PDTC or SB-3CT significantly decreased IL-1β induction of the *MMP-9/TIMP-1* mRNA ratio in the IL-1β + PDTC or WB-3CT + MEL group compared with those in the IL-1β + PDTC and SB-3CT groups (Figure 2M). These results indicate that melatonin affects not only *MMP-9* and *TIMP-1* mRNA levels, but also the *MMP-9/TIMP-1* mRNA ratio.

### Melatonin inhibits MMP-9 activity in pericytes in the BBB model

**Figure 3:**
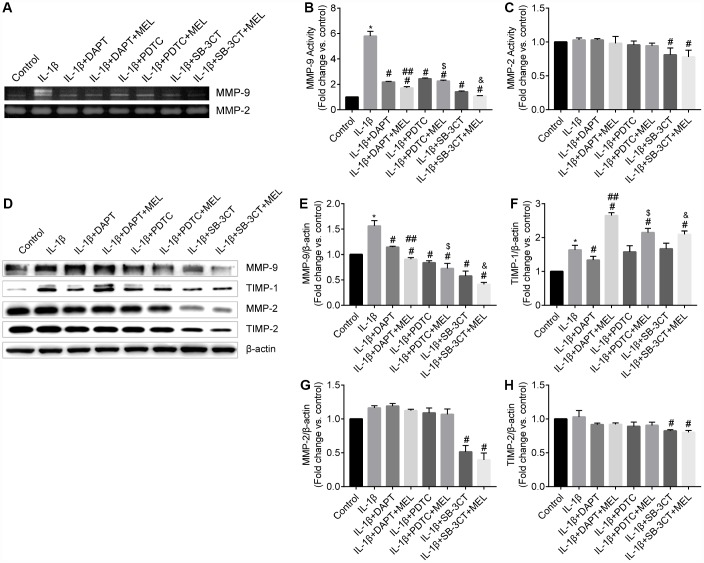
**Melatonin inhibited MMP-9 but not MMP-2 activity and protein levels and increased TIMP-1 but not TIMP-2 protein levels.** (**A**) MMP-9 and MMP-2 activities were measured using gelatin zymography analysis in different groups. (**B**–**C**) MMP-2 and MMP-9 activities were quantified by densitometry analysis. (**D**) MMP-9, MMP-2, TIMP-1, and TIMP-2 protein levels were measured using western blot analysis. (**E**–**H**) MMP-9, MMP-2, TIMP-1, and TIMP-2 protein levels were quantified by densitometry analysis. Results are presented as the mean ± SD, n = 3. ^*^p < 0.01 compared with the control; ^#^*p* < 0.01 compared with the IL-1β-treated group; ^##^*p* < 0.01 compared with the IL-1β + DAPT group; ^$^*p* < 0.01 compared with the IL-1β + PDTC group; ^&^*p* < 0.01 compared with the IL-1β + SB-3CT group.

Gelatin zymography and western blot analysis were used to examine the MMP-2 and MMP-9 enzymatic activities and protein levels (Figure 3A, 3D). MMP-9 enzymatic activity significantly increased in the IL-1β-treated group compared with the control group, but it was suppressed in the IL-1β + DAPT, IL-1β + DAPT + MEL, IL-1β + PDTC, IL-1β + PDTC + MEL, IL-1β + SB-3CT and IL-1β + SB-3CT + MEL groups (Figure 3A, 3B). There was no significant difference in MMP-2 enzymatic activity among the IL-1β-treated, IL-1β + DAPT, IL-1β + DAPT + MEL, IL-1β + PDTC, and IL-1β + PDTC + MEL groups compared with the control group (Figure 3A, 3C). Furthermore, western blot analysis demonstrated that IL-1β significantly increased MMP-9 but not MMP-2 protein levels (Figure 3D, 3E, 3G). Pretreatment with DAPT or PDTC alone or in combination with melatonin inhibited MMP-9 protein expression but had no significant effect on MMP-2 protein level; however, MMP-2 protein level was significantly reduced in the IL-1β + SB-3CT and IL-1β + SB-3CT + MEL groups compared with IL-1β-treated group (Figure 3D, 3G).

Secreted MMPs activity is highly modulated by TIMPs which are the endogenous inhibitors of MMPs. We examined TIMP-1 and TIMP-2 protein levels in pericytes using western blot analysis. When compared with the control group, TIMP-1 was significantly increased in the IL-1β-treated group. However, melatonin pretreatment significantly increased the expression of TIMP-1 protein in the IL-1β + DAPT + Mel group even though DAPT decreased the expression of TIMP-1 in the IL-1β + DAPT group (Figure 3D, 3F). Moreover, there was no difference in TIMP-1 protein level in the IL-1β + PDTC or IL-1β + SB-3CT groups compared with the IL-1β-treated group (Figure 3D, 3F). Changes in the TIMP-2 protein level were only detected in the IL-1β + SB-3CT and IL-1β + SB-3CT + MEL treatment groups, while no changes were detected in the other six groups (Figure 3D, 3H).

### Melatonin inhibits MMP-9-induced BBB permeability

**Figure 4:**
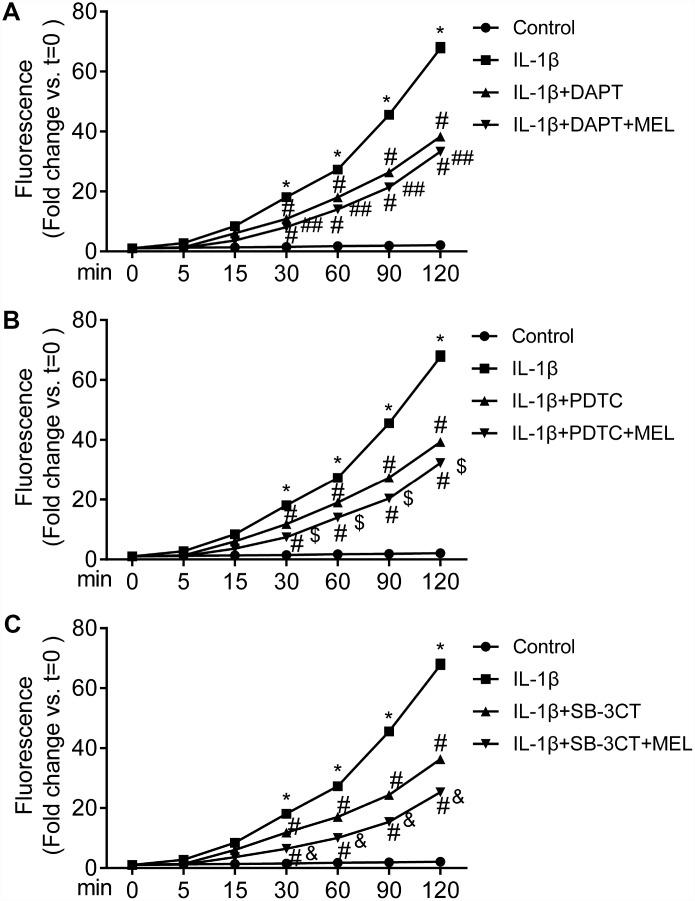
**MMP-9-induced BBB permeability is inhibited by melatonin.** The value of the fluorescence that passed through the inserts was determined at 0, 5, 15, 30, 60, 90 and 120 min. Results are presented as the mean ± SD, n = 3. ^*^p < 0.01 compared with the control; ^#^*p* < 0.01 compared with the IL-1β-treated group; ^##^*p* < 0.01 compared with the IL-1β + DAPT group; ^$^*p* < 0.01 compared with the IL-1β + PDTC group; ^&^*p* < 0.01 compared with the IL-1β + SB-3CT group.

BBB permeability was determined using Na-F in the Transwell assay. The permeability of the BBB in the IL-1β-treated group obviously increased at different time points compared with that in the control group (Figure 4A–4C). However, the permeability to Na-F was significantly reduced in the IL-1β + DAPT, PDTC, and SB-3CT groups relative to the IL-1β-treated group after 30 min of treatment (Figure 4A–4C). Furthermore, the permeability significantly decreased in the IL-1β + DAPT, PDTC, and SB-3CT treatment groups pretreated with MEL relative to the IL-1β + DAPT, PDTC, and SB-3CT groups that did not receive pretreatment (Figure 4A–4C). These results indicate that melatonin can reduce BBB permeability that is induced by IL-1β, which potentially occurs through the stimulation of MMP-9 activity.

### Melatonin inhibits the MMP-9 damage-mediated disruption of adherens and tight junction proteins in BBB

**Figure 5:**
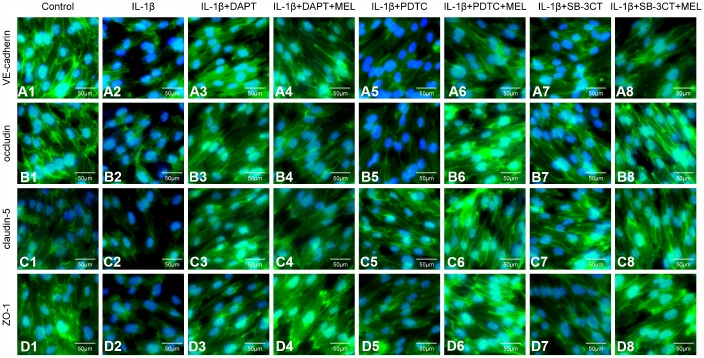
**Melatonin pretreatment ameliorates the effect of MMP-9 on the expression of VE-cadherin, occludin, claudin-5, and ZO-1 in the BBB in different treatment groups.** Cells were treated as described in the Materials and methods for observation by immunofluorescence. (**A1**–**A8**) Immunofluorescence staining of VE-cadherin; (**B1**–**B8**) Immunofluorescence staining of occludin; (**C1**–**C8**) Immunofluorescence staining of claudin-5; (**D1**–**D8**) Immunofluorescence staining of ZO-1.

**Figure 6:**
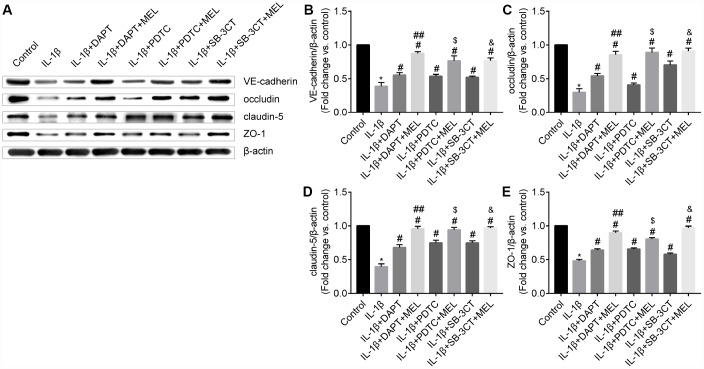
**Melatonin pretreatment reduces the effect of MMP-9 on the expression of VE-cadherin, occludin, claudin-5, and ZO-1 in the BBB.** (**A**) VE-cadherin, occludin, claudin-5 and ZO-1 protein levels were measured using western blot analysis. (**B**–**C**) VE-cadherin, occludin, claudin-5 and ZO-1 protein levels were quantified by densitometry analysis. Results are presented as the mean ± SD, n = 3. ^*^p < 0.01 compared with the control; ^#^*p* < 0.01 compared with the IL-1β-treated group, respectively; ^##^*p* < 0.01 compared with the IL-1β + DAPT group; ^$^*p* < 0.01 compared with the IL-1β + PDTC group; ^&^*p* < 0.01 compared with the IL-1β + SB-3CT group.

MMPs can affect adherens and tight junction proteins, which can influence the integrity of the endothelial barrier. We observed an obvious disruption of adherens junctions (VE-cadherin) and tight junctions (occludin, claudin-5 and ZO-1) in the IL-1β-treated group, in which MMP-9 activities were higher (Figure 5A2–5D2) compared with the control group (Figure 5A1–5D1). However, DAPT, PDTC, and SB-3CT pretreatment attenuated the disruption of VE-cadherin, occludin, claudin-5 and ZO-1 in combination with IL-1β relative to IL-1β treatment alone (Figure 5A3, 5A5, 5A7, 5B3, 5B5, 5B7, 5C3, 5C5, 5C7, 5D3, 5D5, 5D7). Moreover, the IL-1β + DAPT + MEL, or PDTC + MEL, or SB-3CT + MEL groups, which included melatonin pretreatment, had reduced disruptions of VE-cadherin, occludin, claudin-5, and ZO-1 than in IL-1β + DAPT, PDTC, and SB-3CT groups that did not receive the pretreatment (Figure 5A4, 5A6, 5A8, 5B4, 5B6, 5B8, 5C4, 5C6, 5C8, 5D4, 5D6, 5D8). Next, we examined whether increased MMP-9 changed adherens and tight junction protein levels. The protein levels of VE-cadherin, occludin, claudin-5 and ZO-1 in the IL-1β-treated group were decreased by 61.3%, 70.2%, 60.4%, and 51.9%, respectively, while these reductions were largely reversed in IL-1β + DAPT, PDTC, and SB-3CT groups. Moreover, melatonin pretreatment in the IL-1β + DAPT + MEL, PDTC + MEL, SB-3CT + MEL groups was further reversed (Figure 6A–6E). Based on these results, the reduced expression of adherens and tight junction proteins was associated with increased MMP-9 enzymatic activity, while melatonin pretreatment acted against the effect of MMP-9.

### Melatonin inhibits MMP-9 via the NOTCH3-NF-κB pathway

**Figure 7:**
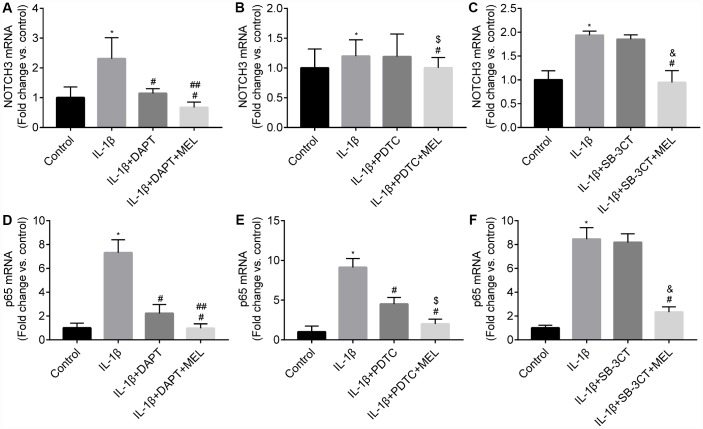
**Melatonin inhibited *NOTCH3* and *p65* gene expression in different groups.** Pericytes in the Transwell were pretreated with IL-1β (10 ng/mL) for 24 h in serum-free medium with or without pretreatment with DAPT (10 μmol/L), PDTC (25 μmol/L l) and WB-3CT (20 μmol/L), and in combination with melatonin (MEL, 10 μmol/L) for 30 min. (**A**–**C**) *NOTCH3* mRNA levels were quantified in IL-1β-treated, IL-1β + DAPT/PDTC/SB-3CT, and IL-1β + DAPT/PDTC/SB-3CT + MEL groups compared with the control group. qRT-PCR data were normalized to *GAPDH* expression. (**D**–**F**) *p65* mRNA levels were quantified in IL-1β-treated, IL-1β + DAPT/PDTC/SB-3CT, and IL-1β + DAPT/PDTC/SB-3CT + MEL groups compared with the control group. Results are presented as the mean ± SD, n = 3. ^*^p < 0.01 compared with the control; ^#^*p* < 0.01 compared with the IL-1β-treated group; ^##^*p* < 0.01 compared with the IL-1β + DAPT group; ^$^*p* < 0.01 compared with the IL-1β + PDTC group; ^&^*p* < 0.01 compared with the IL-1β + SB-3CT group.

We observed *NOTCH3* expression in pericytes and not in astrocytes or endothelial cells (Supplementary Figure 2). The qRT-PCR results showed that the IL-1β-treated group exhibited higher *NOTCH3* expression relative to the control group (Figure 7A–7C). When the NOTHC3 inhibitor DAPT was added, the results showed that *NOTCH3* and *p65* mRNAs were significantly reduced relative to the IL-1β-treated group (Figure 7A, 7D). When melatonin was added to those growth conditions, *NOTCH3* and *p65* expression levels were significantly reduced compared with the IL-1β + DAPT group that did not receive the melatonin pretreatment (Figure 7A, 7D). When the NF-κB inhibitor PDTC was added, *p65* mRNA was significantly reduced relative to IL-1β-treated group (Figure 7E); however, there was no difference in the expression of *NOTCH3* mRNA in the IL-1β + PDTC group relative to IL-1β-treated group (Figure 7B). We hypothesized that NOTCH3 participated in regulating the NF-κB pathway by inducing changes in *NOTCH3* mRNA expression between the IL-1β + DAPT and IL-1β + PDTC groups. When melatonin was added, the results showed that *NOTCH3* and *p65* mRNA were significantly reduced in the IL-1β + PDTC + MEL group compared with the IL-1β + PDTC group (Figure 7B, 7E), which suggested that melatonin could additionally inhibit *NOTCH3* and *p65* expression induced by IL-1β in pericytes. When the MMP-9/-2 inhibitor SB-3CT was added, there was no difference in the expression of *NOTCH3* and *p65* mRNA relative to the IL-1β-treated group (Figure 7C, 7F).

**Figure 8:**
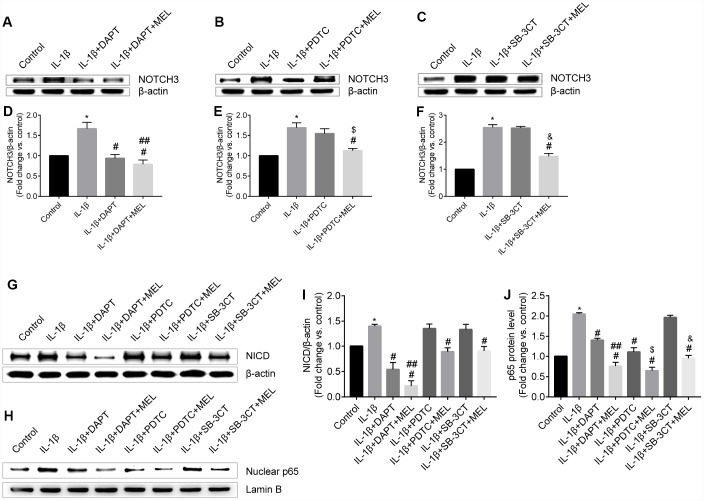
**Melatonin pretreatment affects NOTCH3 and p65 protein expression and nuclear translocation of p65 in the different groups.** (**A**, **D**) NOTCH3 protein expression was analyzed by western blot and quantified by densitometric analysis for IL-1β-treated, IL-1β + DAPT and IL-1β + DAPT + MEL groups compared with the control group; (**B**, **E**) NOTCH3 protein expression was analyzed by western blot and quantified by densitometry analysis in IL-1β-treated, IL-1β + PDTC, IL-1β + PDTC + MEL groups compared with the control group; (**C**, **F**) NOTCH3 protein expression was analyzed by western blot and quantified by densitometry analysis in IL-1β-treated, IL-1β + SB-3CT, and IL-1β + SB-3CT + MEL groups compared with the control group. (**G**, **I**) NICD protein expression was analyzed by western blot and quantified by densitometry analysis in IL-1β-treated, IL-1β + DAPT, IL-1β + DAPT + MEL, IL-1β + PDTC, IL-1β + PDTC + MEL, IL-1β + SB-3CT, and IL-1β + SB-3CT + MEL groups compared with the control group. (**H**, **J**) p65 protein was analyzed by western blot and quantified by densitometry analysis in these eight groups. Results are presented as the mean ± SD, n = 3. ^*^p < 0.01 compared with the control; ^#^*p* < 0.01 compared with the IL-1β-treated group, respectively; ^##^*p* < 0.01 compared with the IL-1β + DAPT group; ^$^*p* < 0.01 compared with the IL-1β + PDTC group; ^&^*p* < 0.01 compared with the IL-1β + SB-3CT group.

The western blot results showed that NOTCH3 increased with IL-1β treatment compared with the control group (Figure 8A, 8D). We observed that NOTCH3 was significantly reduced in the IL-1β + DAPT group relative to the IL-1β-treated group (Figure 8A, 8D). In addition, there was no difference in NOTCH3 expression in the IL-1 β + PDTC group relative to the IL-1β-treated and IL-1β +SB-3CT groups (Figure 8B, 8C, 8E, 8F). We observed similar results in NICD expression among the IL-1β + DAPT, IL-1β + PDTC, and IL-1β +SB-3CT groups relative to the IL-1β-treated group, respectively (Figure 8G, 8I). Therefore, NOTCH3 may regulate the NF-κB signal pathway, rather than vice versa. In addition, melatonin pretreatment inhibited NOTCH3 expression compared with the expression in the IL-1β-treated and IL-1β + DAPT groups, respectively (Figure 8A, 8D). There were similar to results from the IL-1β + PDTC + MEL group compared with the IL-1β-treated and IL-1β + PDTC groups, and in the IL-1β + SB-3CT + MEL group compared with the IL-1β-treated and IL-1β + SB-3CT groups, respectively (Figure 8B, 8C, 8E, 8F).

**Figure 9:**
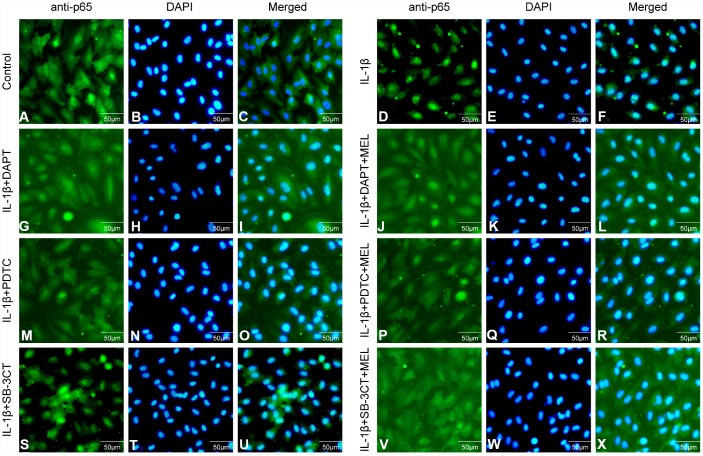
**Effect of melatonin pretreatment on the expression and translocation of p65 in the different groups.** Alexa Fluor 488 conjugated goat anti-p65 (**A**, **D**, **G**, **J**, **M**, **P**, **S**, **V**), DAPI (**B**, **E**, **H**, **K**, **N**, **Q**, **T**, **W**) and merged immunofluorescence (**C**, **F**, **I**, **L**, **O**, **R**, **U**, **x**) were observed under a fluorescence microscope.

**Figure 10:**
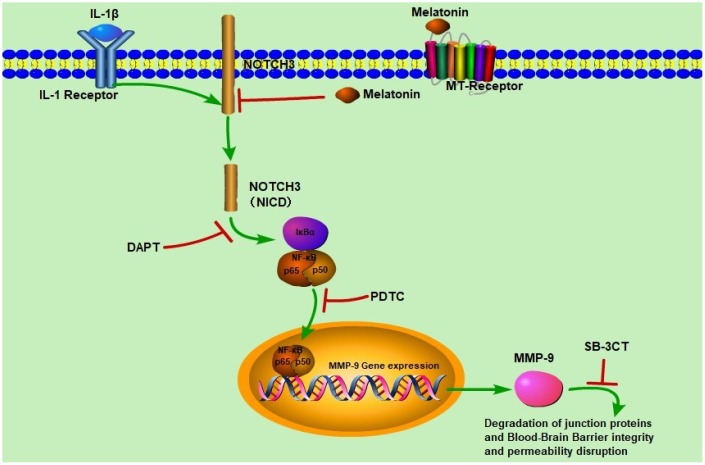
**Schematic diagram of the regulatory effects of melatonin on BBB integrity and permeability.** IL-1β induced NOTCH3 (NICD) expression which activated NF-κB/p65 expression. NF-κB/p65 translocated to the nucleus to induce *MMP-9* expression, and MMP-9 disrupted BBB integrity and permeability. When melatonin, DAPT, PDTC, and SB-3CT were added respectively, NOTCH3 expression was significantly inhibited, leading to decreased NF-κB/p65 and MMP-9 expression, which protected BBB integrity and permeability.

NF-κB/p65 is a member of the NF-κB transcription factor family. These transcription factors translocate to the nucleus and bind to a specific sequence in the genome to regulate MMPs transcription when cells are stimulated with inflammatory factors. We observed that activated NF-κB/p65 increased in the IL-1β-treated group, while pretreatment with DAPT or PDTC inhibited the nuclear translocation of NF-κB/p65 relative to IL-1 β treatment alone (Figure 8H, 8J). However, there was no difference in the IL-1β + SB-3CT group relative to the IL-1 β-treated group (Figure 8, 8H, 8J). Next, we also analyzed the cellular location changes of NF-κB/p65 after IL-1 β treatment, pretreated with DAPT, PDTC, and SB-3CT in combination with melatonin by immunostaining method. We observed that NF-κB/p65 was located in the cytoplasm in the control group (Figure 9A–9C), and translocated into the nucleus in IL-1β-treated cells (Figure 9D–9F). Pretreatment with DAPT and PDTC inhibited the p65 translocation, indicated by changes in immunofluorescence patterns in cells from the IL-1β + DAPT and IL-1β + PDTC groups (Figure 9G–9I, 9M–9O). This inhibition was not observed in the IL-1β + SB-3CT group (Figure 9S–9U). Pretreatment with melatonin clearly suppressed the p65 translocation, as seen in the IL-1β + DAPT + MEL, IL-1β + PDTC + MEL and IL-1β + SB-3CT + MEL groups (Figure 9J–9L, 9P–9R, 9V–9x). Taken together, these results demonstrated that melatonin protects BBB integrity and permeability via the NOTCH3/NF-κB pathway (Figure 10).

## DISCUSSION

The results of our study can be summarized as follows. The expression levels of MMP-9 mRNA and protein were upregulated in pericytes upon IL-1β stimulation, and the mRNA and protein levels of the endogenous inhibitor *TIMP-1* were also upregulated, which increased the MMP-9/TIMP-1 ratio. Conversely, changes in MMP-2 mRNA and protein levels in response to IL-1β stimulation were not observed. Second, melatonin pretreatment decreased the expression levels of MMP-9 mRNA and protein but increased TIMP-1 mRNA and protein levels. Third, increased MMP-9 activity disrupted adherens and tight junctions of the BBB, and increased endothelial cell permeability to Na-F. Fourth, melatonin decreased the MMP-9/TIMP-1 ratio and reversed the damaging effect of MMP-9 had on endothelial cell-cell junctions, providing a safeguard for BBB integrity. Finally, melatonin inhibited NOTCH3, prevented the nuclear translocation of p65, and further reduced *MMP-9* gene expression. To our knowledge, these results are the first to demonstrate that IL-1β-induced MMP-9 expression and activity in pericytes limits the disruption of BBB integrity.

MMPs and endogenous TIMPs are important regulators and play key roles in many biological processes and diseases [50]. The role of MMP-9 secreted from pericytes in regulating BBB integrity is not yet well elucidated. In order to investigate this role, we analyzed *MMP-9/-2* and *TIMP-1/-2* expression in pericytes by qRT-PCR. The results suggested that MMP-9 mRNA level was increased in IL-1β-treated pericytes and was reduced with pretreatments of SB-3CT, PDTC, DAPT. In addition, in combination with melatonin, these inhibitors initiated a further reduction in *MMP-9* mRNA and protein but increased *TIMP-1* mRNA and protein levels. Tai *et al* [54] demonstrated that melatonin decreased neurovascular damage by attenuating MMP-9 activation and increasing TIMP-1 in transient focal cerebral ischemia. We also analyzed MMP-9 and MMP-2 activities in pericytes by gelatin zymography, which indicated that MMP-9 increased in IL-1β-treated pericytes. When the inhibitors SB-3CT, PDTC, and DAPT were added to the cultures in the presence of IL-1β, MMP-9 activity significantly decreased to varying degrees. In addition, SB-3CT, or PDTC, or DAPT in combination with melatonin pretreatment significantly attenuated MMP-9 activity, suggesting that melatonin is a potential MMP-9 inhibitor [55].

The BBB contains an important endothelial cell barrier structure that plays an important role in the maintenance of CNS homeostasis [50, 56]. The fundamental basis for barrier function mainly consists of adherens and tight junctions in endothelial cells, which seal off the paracellular pathway between adjacent brain capillary endothelial cells [57, 58]. VE-cadherin is the main adherens junction in endothelial cells of all types of vessels [58]. The main tight junction proteins include occludin, claudins, and ZO-1 [56]. Therefore, maintaining and regulating BBB functions are important to assure the integrity of the VE-cadherin adherens junction protein and the tight junction proteins occludin, claudin-5, and ZO-1, which depend on the extent of the endothelial cells barrier permeability [50, 59]. Pericytes are critical coordinators of BBB functions and CNS homeostasis, regulate cerebral blood flow, contribute to the secretion of ECM proteins, and provide structural support and angioarchitecture [59]. The BBB endothelial permeability changes involve the activation of MMPs and spatial redistribution of junction proteins [60]. In the BBB, MMP-9 is primarily secreted from pericytes and is associated with BBB disruption in several diseases including ischemic stroke, intracerebral hemorrhage, hemorrhagic transformation, brain edema, Alzheimer’s disease and Parkinson’s disease [16, 61]. Furthermore, oxidants, inflammatory cytokines, and pharmacological agents can provoke MMPs activations that disrupts the adherens junction and the tight junction proteins [50]. Our previous studies suggested that IL-1β increased the permeability of the HUVEC monolayer by disrupting adherens and tight junctions and expanding the junction gap widths [50, 62]. In this study, we showed that in IL-1β-induced pericytes, there was a significant increase in MMP-9, which enlarged the intercellular gap by destroying the cell junction elements VE-cadherin, occludin, claudin-5, and ZO-1; this was consistent with the observed increase in BBB permeability to Na-F. When the MMP-9/-2 inhibitor WB-3CT was added, the results showed that the BBB permeability was decreased. Moreover, when the NF-κB inhibitor PDTC was added, the BBB permeability decreased, as well as the expression of *MMP-9* and the *MMP-9/TIMP-1* ratio; however, no changes were observed in the expression of *MMP-2* and *NOTCH3*. Furthermore, when the NOTCH inhibitor DAPT was added, the BBB permeability also decreased and *MMP-9* expression, the *MMP-9/TIMP-1* ratio, and *p65* expression were reduced, while no changes occurred in MMP-2 expression.

The BBB permeability due to hyperglycemia during early reperfusion in stroke is involved in autophagy- and MMP-2/9-mediated reduction and redistribution of tight junction proteins [63]. The BBB properties were substantially induced by brain pericytes through the change of the expression of tight junction proteins and Na-F permeability. In addition, limited primary cell availability essentially eliminates the possibility of using patient-matched brain pericytes for disease modeling applications. Stebbins *et al* [27] demonstrated that hPSC-derived brain pericyte-like cells in the BBB were indistinguishable from those induced by primary human brain pericytes. Pericyte involvement in CADASIL can result in increased capillary vessels permeability and disturbances in cerebral microcirculation, which can lead to white matter injury. Since capillary pericytes regulate vessel contractility, their degeneration can also cause defective vasomotor reactivity, a phenomenon observed very early in CADASIL, before the development of histopathological changes in vessel walls [23]. Interestingly, following white matter demyelination, pericytes promote differentiation of oligodendrocyte progenitors involved in CNS regeneration via the a2-chain of laminin [64]. Moreover, pericytes are involved in the recruitment of neutrophils to the sub-endothelial layer and breaching of the vascular wall by secreting the chemoattractant IL-8 and the metalloproteinases MMP-2 and MMP-9, which break down endothelial cell-to-cell contacts [65]. Pericyte degeneration also causes the breakdown of the BBB, leading to blood-derived toxic substances passing into the central nervous system [6]. As a crucial component of the neurovascular unit, pericytes have diverse functions that regulate capillary blood flow and BBB permeability, as well as BBB stability [66]. Indeed, pericyte-deficient animals do not establish proper vessel boundaries and ultimately cause BBB breakdown and leakage [67]. BBB dysfunction results from tight junction changes, increased transcytosis, loss of trophic support from pericytes or altered hemodynamics [68].

Melatonin is mainly secreted by the pineal gland [51, 52] and has powerful antioxidant effects, including strong free-radical scavenging that can regulate several molecular pathways under different pathological conditions, including oxidative stress [69], inflammation, apoptosis, and cell death [70]. Melatonin has direct and indirect protective functions through the detoxification of free radicals, mitochondrial protection, reduction of free radical formation, prevention of neuronal overexcitation and several anti-inflammatory actions in neurodegenerative diseases, such as Alzheimer’s disease, Huntington’ disease, Parkinson’s disease, amyotrophic lateral sclerosis, spinocerebellar ataxia, and multiple sclerosis [70]. Melatonin plays a regulatory role in MMP-mediated physiological and pathological processes and human diseases. Melatonin can inhibit the tumorigenesis of bladder cancer and renal cell carcinoma metastasis by downregulating MMP-9 expression through different signaling pathways [71, 72]. In our study, melatonin pretreatment of the IL-1β + DAPT/PDTC/SB-3CT + MEL groups inhibited the increase of MMP-9, reversed the MMP-9-induced damage of VE-cadherin, occludin, claudin-5, and ZO-1, and reduced the increase in BBB permeability to Na-F. In addition, melatonin promotes the expression of the MMP-9 endogenous inhibitor *TIMP-1* and decreases the *MMP-9/TIMP-1* ratio compared with that in the IL-1β-treated and IL-1β + DAPT/PDTC/SB-3CT groups. These results suggest melatonin could counter the damaging effects of MMP-9 by modulating the regulatory balance between MMP-9 and TIMP-1.

Many recent studies suggested that inflammatory factors (TNF-α or IL-1β) or medicines can inhibit MMP-9 expression via the NF-κB signaling pathway in different types of cells in different diseases [73–76]. In our study, when pericytes cultures were pretreated with the NF-κB inhibitor PDTC, the expression of MMP-9 decreased by PDTC indicating MMP-9 expression is regulated by the NF-κB signaling pathway. Activation of NF-κB by NOTCH3 led to T-cell tumorigenesis and enhanced NF-κB-dependent anti-apoptotic and proliferative pathways [77]. NOTCH3 down-regulation by siRNA decreased the expression of MMP-9 in the hepatocellular carcinoma [78]. When pericytes cells from the BBB *in vitro* model were pretreated with the NF-κB inhibitor PDTC, NOTCH3 expression was unaffected. However, pretreatment with the NOTCH3 inhibitor DAPT significantly inhibited the expression of NF-κB/p65 and translocation of NF-κB/p65 to the nucleus, which suggests that the NF-κB signaling pathway is regulated by NOTCH3. If the expression of NOTCH3 was significantly increased, it could also increase the expression of MMP-9, which damages the intact of BBB. This interpretation is consistent with our experimental results. How pericytes are causally involved in neuroinflammation will improve our understanding of how infection alters the bidirectional crosstalk that modulates brain functions in the neurovascular unit. The NOTCH3 research field is still in its infancy and we need more evidence of the impact of NOTCH3 in pericytes on the vascular integrity of the BBB. Moreover, we are particularly interested in further investigations into the role of NOTCH3 in pericytes and how it contributes to the integrity and function of the BBB in animal models or clinical studies.

In conclusion, the results from our study demonstrate an aggregation-dependent effect of IL-1β on the increased expression of NOTCH3 in pericytes, which can induce MMP-9 secretion from pericytes and disrupt BBB integrity. Moreover, our study highlighted MMP-9 as one of the mediators of IL-1β-induced alteration of NOTCH3. These results are consistent with the hypothesis that MMP-9 is involved in BBB pathogenesis through potential NOTCH3/NF-κB signaling pathways. We also suggest that pericytes are potential participants in the events of the BBB pathogenesis in CSVD.

## MATERIALS AND METHODS

### Cell cultures

Primary human brain microvascular pericytes, endothelial cells, and astrocytes were obtained from ScienCell (Carlsbad, CA, USA). Pericytes were maintained in pericyte culture media (ScienCell, Carlsbad, CA, USA) supplemented with 2% fetal bovine serum, pericyte growth supplement (ScienCell), and 1% penicillin/streptomycin(ScienCell, Carlsbad, CA, USA). Endothelial cells were maintained in endothelial culture media (ScienCell, Carlsbad, CA, USA) supplemented with 5% fetal bovine serum, endothelial cell growth supplement (ScienCell), and 1% penicillin/streptomycin (ScienCell, Carlsbad, CA, USA). Astrocytes were maintained in astrocyte culture media (ScienCell, Carlsbad, CA, USA) supplemented with 2% fetal bovine serum, astrocyte growth supplement (ScienCell, Carlsbad, CA, USA), and 1% penicillin/streptomycin (ScienCell, Carlsbad, CA, USA). Cells were cultured in humidified air containing 5% CO_2_ (v/v) at 37°C in an incubator and used at passage 2 for all experiments.

### Construction of the in vitro BBB model [79]

The schematic drawing of the *in vitro* BBB model is presented in Supplementary Figure 3. On the day the endothelial cells were plated, the *in vitro* model was established and defined as day zero. To construct the *in vitro* BBB model, pericytes and astrocytes (1.5 × 10^4^ cells/cm^2^) were seeded on the bottom side of a collagen-coated polyester membrane of Transwell inserts and on the bottom side of the collagen-coated polyester membrane of the Transwell (Corning Life Sciences, MA, USA). The cells were allowed to adhere firmly for overnight, then endothelial cells (1.5 × 10^5^ cells/cm^2^) were seeded on the upper side of the inserts placed in the well of the 6-well culture plates. From day 1, the BBB model was maintained in endothelial culture media, and the *in vitro* BBB model was established within 3 days after setting of the cells.

### Cell treatment conditions

In order to examine the levels of MMPs secreted by pericytes, the BBB model cells were incubated in serum-free medium on the lower side of Transwell inserts containing 10 ng/mL IL-1β (w/v) with or without DAPT (10 μmol/L), PDTC (25 μmol/L), or SB-3CT (20 μmol/L) and with or without melatonin (MEL, 10 μmol/L) pretreatment for 24 h after the cells reached confluency. The BBB model was divided into eight groups: (i) the control group, which was incubated in serum-free medium for 24 h; (ii) IL-1β-treated group, in which cells were incubated in the presence of 10 ng/mL IL-1β for 24 h; (iii) IL-1β + DAPT group, which included cells that were pretreated in 10 μmol/L DAPT for 30 min, before 10 ng/mL IL-1β was added for 24 h (10 μmol/L DAPT pretreatment for 30 min + 10 ng/mL IL-1β); (iv) IL-1β + DAPT + MEL group, in which cells were pretreated with 10 μmol/L melatonin for 30 min, then with10 μmol/L DAPT for 30 min before adding 10 ng/mL IL-1β for 24 h (10 μmol/L melatonin pretreatment for 30 min + 10 μmol/L DAPT pretreatment for 30 min + 10 ng/mL IL-1β); (v) IL-1β + PDTC group, in which cells were pretreated with 25 μmol/L PDTC for 30 min before adding 10 ng/ml IL-1β for 24 h (25 μmol/L PDTC pretreatment for 30 min + 10 ng/mL IL-1β); (vi) IL-1β + PDTC + MEL group, included cells pretreated with 10 μmol/L melatonin for 30 min, and then with 25 μmol/L PDTC for 30 min before adding 10 ng/mL IL-1β for 24 h (25 μmol/L PDTC pretreatment for 30 min + 10 μmol/L melatonin pretreatment for 30 min + 10 ng/mL IL-1β); (vii) IL-1β + SB-3CT group, which included cells pretreated with 20 μmol/L SB-3CT for 30 min and then 10 ng/mL IL-1β for 24 h (20 μmol/L SB-3CT pretreatment for 30 min + 10 ng/mL IL-1β); and (viii) IL-1β + SB-3CT + MEL group, in which cells received pretreatments of 10 μmol/L melatonin for 30 min and 20 μmol/L SB-3CT for 30 min before being cultured in 10 ng/mL IL-1β for 24 h (10 μmol/L melatonin pretreatment for 30 min + 20 μmol/L SB-3CT pretreatment for 30 min + 10 ng/mL IL-1β). Cell culture supernatants were collected after 24 h and stored at -80°C until further experimentation.

### RNA extraction and quantitative real-time PCR (qRT-PCR) analysis

Total RNA was extracted from the pericytes of BBB model cells using the TRIzol reagent (Invitrogen, USA) according to the manufacturer’s protocol and treated with DNase I (Ambion, USA) at 37°C for 30 min. Reverse transcription was performed using a Reverse Transcription kit (Takara, Japan) according to the manufacturer’s instructions. The qRT-PCR was performed with the Hieff^TM^ qPCR SYBR^®^ Green Master Mix (Yeasen, Shanghai, China) using a Step One Plus Real-Time PCR Detection System (Applied Biosystems, USA). Relative expression of mRNA was evaluated using the 2^−ΔΔCt^ method and normalized to the expression of *GAPDH*. Supplementary Table 1 shows the primers used for amplification of the genes that were analyzed.

### Evaluation of blood-brain barrier permeability

The flux of sodium fluorescein (Na-F) across the endothelial monolayers in a Transwell system (0.4 μm pore size polyester membrane inserts, Corning, Union City, CA, USA) was determined as previously described [50, 79]. When all cells reached confluency, the BBB model cells were treated with IL-1β (10 ng/mL) with or without DAPT (10 μmol/L), or PDTC (25 μmol/L), or SB-3CT (20 μmol/L), and with or without melatonin (MEL, 10 μmol/L) in serum-free medium for 24 h. Briefly, 10 μg/mL Na-F (MW: 376 Da) was added to the upper inserts of the Transwell system for 0, 5, 15, 30, 60, 90, and 120 min, at which time 10 μL medium was aspirated from the lower compartments and replaced with the same volume of fresh serum-free medium. The fluorescence value that passed through the inserts was determined by fluorescence multiwall plate reader Synergy4 (BioTek, USA) at an excitation wavelength of 485 nm, and an emission wavelength of 535 nm.

### Gelatin zymography

To assay the enzymatic activity of MMP-2 and MMP-9, conditioned media from the BBB models were analyzed by gelatin zymography [50, 80, 81]. Serum-free conditioned medias were subjected to 10% sodium dodecyl sulfate-polyacrylamide gels (SDS-PAGE) containing 1mg/mL gelatin (Sigma-Aldrich, St. Louis, MO, USA) in non-reducing conditions. Protein samples were mixed with 1% (w/v) SDS sample buffer in non-reducing conditions and loaded onto the gels and ran at 125 V until the Bromophenol Blue dye ran off the end of the gels. Gels were washed twice with 2.5% (v/v) Triton X-100 and subsequently incubated in developing buffer containing 50mmol/L Tris/HCl (pH 7.8), 0.2 mol/L NaCl, 5 mmol/L CaCl2 and 0.02% sodium azide for 42 h at 37°C. Gels were stained with 0.1% Coomassie Blue R-250 followed by destaining in different concentrations of methanol/acetic acid (30% (v/v)/ 10% (v/v), 30 min; 20% (v/v)/ 10% (v/v) for 1 h, and 10% (v/v)/ 5% (v/v), 2 h). Gelatinolytic activity appeared as transparent bands on a blue background and was quantified by scanning the bands using an automatic chemiluminescence image analysis system (Tanon-5200, Shanghai, China). The band intensities were quantified using the ImageJ program (https://rsb.info.nih.gov/ij).

### Western blot analysis

Total proteins (NOTCH3, MMP-9, MMP-2, TIMP-1, TIMP-2, and NF-κB/p65 proteins from pericytes; VE-cadherin, claudin-5, occludin, and ZO-1 proteins from endothelial cells) were isolated from BBB model cells lysed with RIPA lysis buffer containing a protease inhibitor cocktail (Beyotime, China) and were quantified using a BCA Protein Assay kit (Beyotime, China). The nuclear proteins were extracted using NE-PER^®^ Nuclear and Cytoplasmic Extraction Reagents (Thermo Scientific, Waltham, MA, USA) and quantified with a BCA Protein Assay kit (Beyotime, China). Protein fractions were separated by SDS-PAGE, and then transferred to 0.45 μm PVDF membranes (Millipore, USA). The membranes were blocked with 5% non-fat milk in TBST for 1 h and then incubated with specific primary antibodies (anti-MMP-9, anti-MMP-2, anti-TIMP-1, anti-TIMP-2, anti-claudin-5, anti-ZO-1, anti-VE-cadherin and anti-NF-κB/p65 rabbit polyclonal antibody, anti-occludin rabbit monoclonal antibody, all from Abcam, Cambridge, MA; anti-NOTCH3 mouse monoclonal antibody from Santa Cruz, CA, USA) overnight at 4 °C. Horseradish peroxidase-conjugated anti-mouse (Santa Cruz, CA, USA), anti-rabbit immunoglobulins (Abcam, Cambridge, MA) were applied as secondary antibodies for 1h. Bands were visualized using a chemiluminescence ECL reagent (Millipore, USA) and scanned with an automatic chemiluminescence image analysis system (Tanon-5200, Shanghai, China). The band intensities were quantified using the ImageJ program (https://rsb.info.nih.gov/ij).

### Immunofluorescence staining

To characterize the cultures after washing and fixation, brain pericytes were incubated with anti-α-smooth muscle actin (α-SMA) mouse monoclonal antibody (Abcam, ab7817) and anti- neuron-glial antigen 2 (NG2) rabbit polyclonal antibody (Abcam, ab129051), endothelial cells with anti-von Willebrand factor (vWF) rabbit polyclonal antibody (Abcam, ab6994), and astrocytes with anti-GFAP rabbit polyclonal antibody(Abcam, ab7260). All primary antibodies were used in a dilution according to the manufacturer instructions. Secondary antibodies Alexa Fluor 488 conjugated goat anti-mouse and anti-rabbit immunoglobulins H&L (Abcam, ab150113, ab150077) were also used in dilutions according to the manufacturer instructions. 4, 6-diamidino-2-phenylindole (DAPI) was used according to the manufacturer instructions to counterstain cell nuclei. To stain junction proteins, BBB endothelial cells from each of eight groups cultured on the upper side of the inserts were stained for vascular endothelial cadherin (VE-cadherin), occludin, claudin-5, and ZO-1 (Abcam; ab33168, ab216327, and ab15106, ab96587, respectively). The cultures were washed in PBS and fixed with ethanol for 30 min at 4 °C (occludin and claudin-5), 95% ethanol: 5% acetic acid for 10 min at -20 °C (ZO-1), or with 4% paraformaldehyde in PBS for 15 min and then permeabilized for 10 min in 0.1% Triton X-100 at room temperature (VE-cadherin and NF-κB/p65). Cells were blocked with 3% BSA and incubated with anti-occludin, anti-claudin-5, anti-ZO-1, anti-VE-cadherin and anti-NF-κB/p65 (Abcam, ab16502) primary antibodies overnight. A 1-h long incubation with Alexa Fluor 488-conjugated goat anti-rabbit immunoglobulins H&L secondary antibody (Abcam, ab150077) was conducted at room temperature. Cells were washed three times with PBS between each incubation. Images were acquired by fluorescence microscopy (Olympus Corporation, Monolith, Japan) using appropriate filter settings.

### Statistical analysis

All data are presented as the mean ± SD. The statistical analyses were performed using GraphPad Prism 7.0 (GraphPad, San Diego, CA, USA). The statistical significance of the differences between groups was assessed by one-way analysis of variance (ANOVA) for factorial comparisons. Multiple comparisons were also performed using Dunnett’s test. Comparisons between two groups were performed using the Student’s unpaired t-test and Welch’s t-test in the case of equal and unequal variances for the two groups, respectively. Differences with a *p*-value < 0.05 were considered statistically significant. All experiments were repeated at least three times.

## Supplementary Material

Supplementary Figures

Supplementary Table 1
