# Supplementary material for: Melatonin protects blood-brain barrier integrity and permeability by inhibiting matrix metalloproteinase-9 via the NOTCH3/NF-κB pathway
Source: Aging (Albany NY). 2019 Dec 7;11(23):11391–415. doi: 10.18632/aging.102537 (PMC6932927; doi:10.18632/aging.102537)
Supplement: Supplementary Figures [file aging-11-102537-s002..pdf]

SUPPLEMENTARY FIGURES

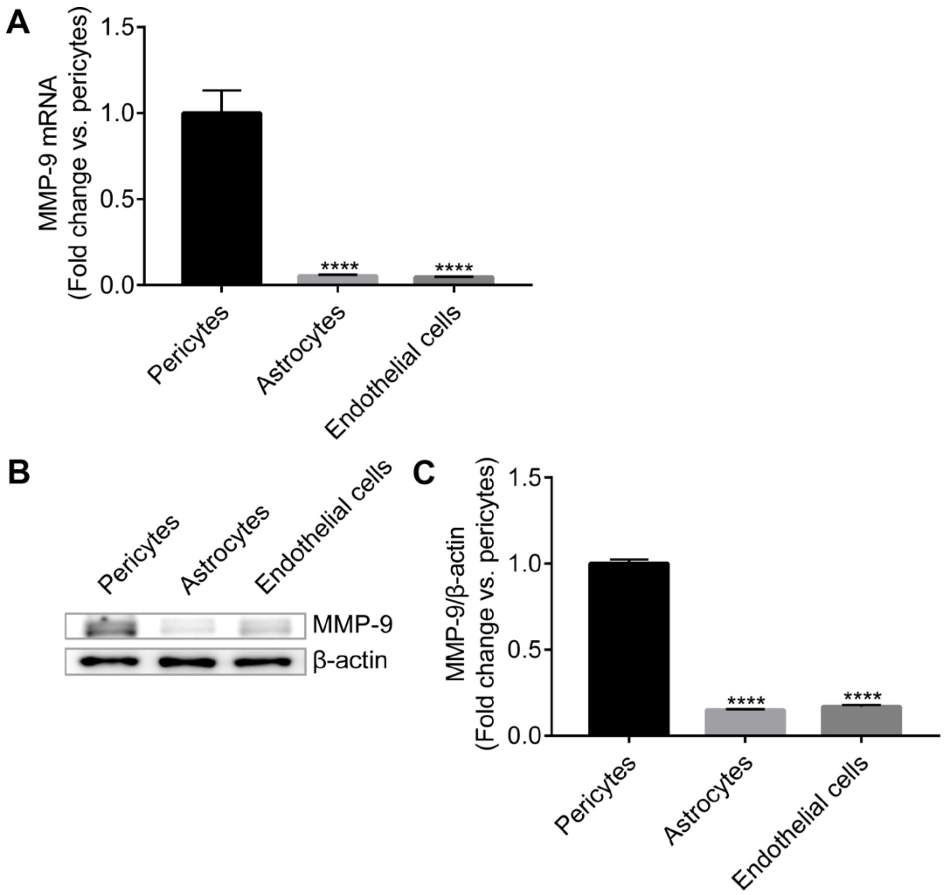

Supplementary Figure 1. Pericytes were the primary producer of MMP-9, rather than astrocytes and endothelial cells with the IL-1β induction. (**A**) qRT-PCR analysis of the expression levels of *MMP-9* in pericytes, astrocytes and endothelial cells from BBB model cells. The data, normalized to *GAPDH* expression, are presented as the mean ± SD, n=3. $^{****}p < 0.0001$ compared with pericytes. (**B**) Western blot analysis of the expression levels of MMP-9 in pericytes, astrocytes and endothelial cells from BBB model cells. (**C**) MMP-9 protein level was quantified by densitometry analysis using ImageJ. The data, normalized to β-actin expression, are presented as the mean ± SD, n=3. $^{****}p < 0.0001$ compared with pericytes.

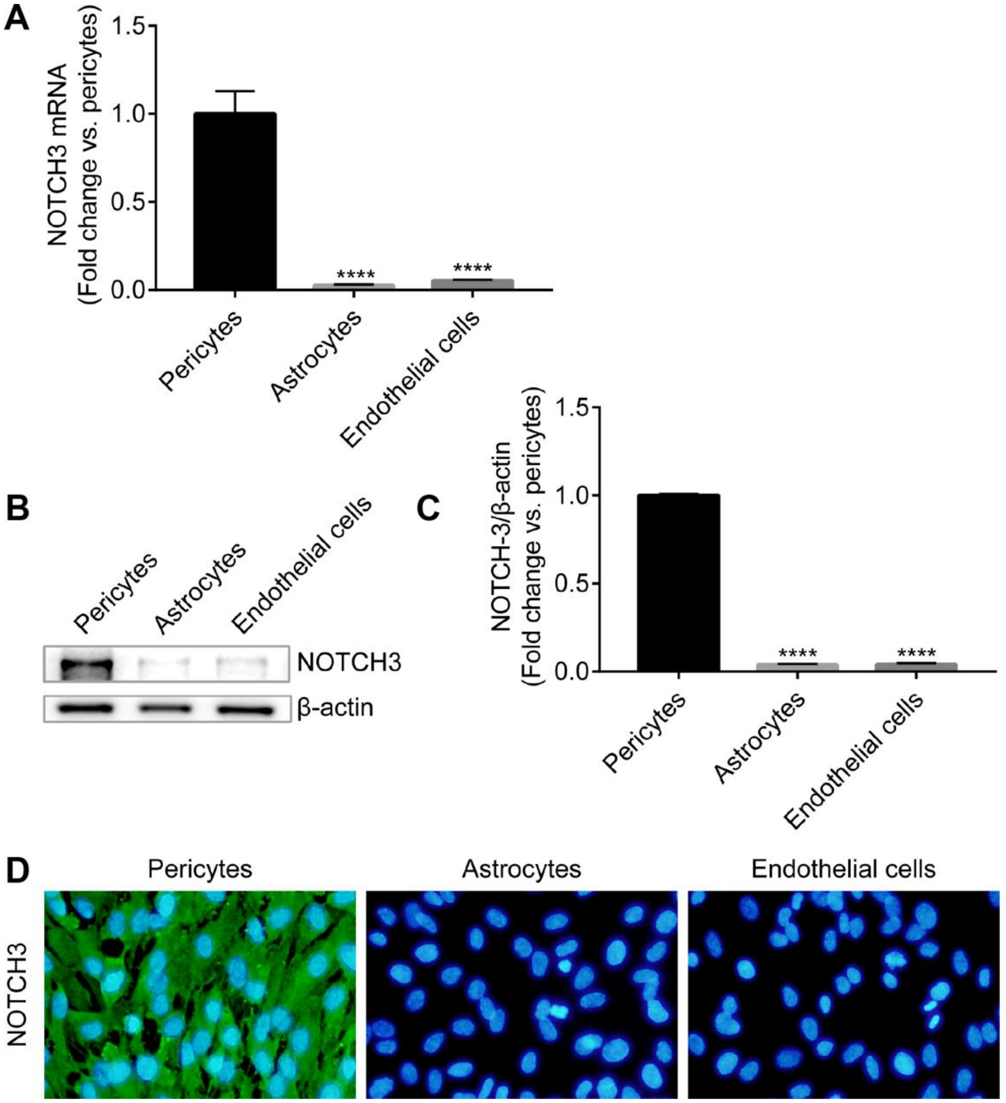

Supplementary Figure 2. The expression of NOTCH3 in the pericytes, not in the astrocytes and endothelial cells under the IL-1β induction. (**A**) qRT-PCR analysis of the expression levels of *NOTCH3* in pericytes, astrocytes and endothelial cells from BBB model cells. The data, normalized to *GAPDH* expression, are presented as the mean ± SD, n=3. $^{****}p < 0.0001$ compared with pericytes. (**B**) Western blot analysis of the expression levels of NOTCH3 in pericytes, astrocytes and endothelial cells from BBB model cells. (**C**) NOTCH3 protein level was quantified by densitometry analysis using ImageJ. The data, normalized to β-actin expression, are presented as the mean ± SD, n=3. $^{****}p <$ 0.0001 compared with pericytes. (**D**) Pericytes show positive immunostaining for NOTCH3, while endothelial cells and astrocytes are negative for NOTCH3 by immunofluorescence microscopy. Bar = 50 µm.

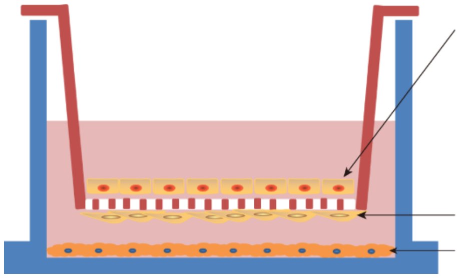

Supplementary Figure 3. The schematic drawing of the *in vitro* BBB model. Human brain endothelial cells are seeded to the luminal compartment of the inserts and pericytes are seeded on the other side, and astrocytes are seeded at the bottom of 6-well Transwell.
