# Supplementary material for: Melatonin protects blood-brain barrier integrity and permeability by inhibiting matrix metalloproteinase-9 via the NOTCH3/NF-κB pathway
Source: Aging (Albany NY). 2019 Dec 7;11(23):11391–415. doi: 10.18632/aging.102537 (PMC6932927; doi:10.18632/aging.102537)
Supplement: Supplementary Table 1 [file aging-11-102537-s001..pdf]

SUPPLEMENTARY TABLE

Supplementary Table 1. The primers of related genes.

Gene name	Forward sequence (5′-3′)	Reverse sequence (5′-3′)
MMP-9	AGACCTGGGCAGATTCCAAAC	CGGCAAGTCTTCCGAGTAGT
MMP-2	TACAGGATCATTGGCTACACACC	GGTCACATCGCTCCAGACT
TIMP-1	CTTCTGCAATTCCGACCTCGT	ACGCTGGTATAAGGTGGTCTG
TIMP-2	GCTGCGAGTGCAAGATCAC	TGGTGCCCGTTGATGTTCTTC
NOTCH3	TGGCGACCTCACTTACGACT	CACTGGCAGTTATAGGTGTTGAC
NF-κB/p65	GTGCAGAAAGAAGACATTGA	AGGCTAGGGTCAGCGTATGG
GAPDH	ACAACTTTGGTATCGTGGAAGG	GCCATCACGCCACAGTTTC
